# Aerobic Oxidation of 5-Hydroxymethylfurfural over Ag Nanoparticle Catalysts Stabilized by Polyvinylpyrrolidone with Different Molecular Weights

**DOI:** 10.3390/nano10091624

**Published:** 2020-08-19

**Authors:** Haian Xia, Jiahuan An, Weizi Zhang

**Affiliations:** 1 Jiangsu Provincial Key Lab for the Chemistry and Utilization of Agro-forest Biomass, College of Chemical Engineering, Nanjing Forestry University, Nanjing 210037, China; jiahuanan11@outlook.com (J.A.); zhangwz@njfu.edu.cn (W.Z.); 2 Jiangsu Co-Innovation Center of Efficient Processing and Utilization of Forest Resources, Nanjing Forestry University, Nanjing 210037, China

**Keywords:** metal–support interaction, molecular weight, capping agent, PVP, HMF oxidation

## Abstract

The metal–support interaction (MSI) has a remarkable effect on the catalytic properties, but how to precisely modulate its degree remains a huge challenge. Herein, polyvinylpyrrolidone (PVP) with three different molecular weights (MWs) (24, 58, and 130 kDa) was used as a capping agent to fabricate Ag nanoparticles (NPs) supported on ZrO_2_. The physiochemical properties of the catalysts were characterized by X-ray diffraction (XRD), Transmission Electron Microscope (TEM), X-ray Photoelectron Spectroscopy (XPS), and Fourier transform infrared (FT-IR) techniques. The impacts of MSI on the catalytic activity and reaction kinetics for aerobic oxidation of 5-hydroxymethylfurfural (HMF) were investigated. The results showed that the introduction of PVP with various MWs could efficiently tailor the interfacial interactions and charge transfers (CT) among PVP, the support, and Ag NPs, thereby affecting the oxidation activity of HMF. The turnover number (TON) for HMF oxidation decreases in the order of unsupported colloidal Ag clusters > Ag/ZrO_2_ (58,000) > Ag/ZrO_2_ (130,000) > Ag/ZrO_2_ (24,000) > Ag/ZrO_2_. The reason for this large difference in the catalytic activity for HMF oxidation is that various MWs of PVP result in a change of MSI, thereby facilitating CT from PVP to Ag metal sites. This study offers a new strategy for modulating MSI by varying the MW of capping agents, thereby tuning the catalytic properties in the oxidation of HMF.

## 1. Introduction

The catalytic activity of metal nanoparticles (NPs) highly depends on their particle size, shape, and surface composition [1,2,3]. Additionally, metal–support interaction (MSI) has significant impacts on catalytic properties due to electronic effect, geometric effects, and so on [1,4,5,6,7]. The classic strong metal–support interaction (SMSI) refers to reducible oxide supports (i.e., TiO_2_, CeO_2_) covering metal NPs to form a thin oxide overlayer [7]. However, the precise modulation of metal–support interactions remains a tremendous challenge in heterogeneous catalysis, although some advances have been achieved in the field in the past few years.

Colloidal metal NPs have attracted considerable attention, because the usage of organic ligands (often referred to as stabilizers and capping agents) can precisely control particle size, shape, and surface composition [2]. Polyvinylpyrrolidone (PVP) may act as a capping/stabilizing agent and an electron donor to control the NP size [2,8,9,10]. It has been reported that capping agents have either beneficial effects or negative influences on the catalytic activity, which is highly dependent on the reaction types [2,9]. Skurai et al. investigated the impact of PVP chain length on the catalytic activities of Au nanoclusters capped by PVP [11]. They revealed that long-chain PVP results in highly entangled structures through entanglement of the Au nanocluster surface, which increases the electronegativity at the cluster’s surface [11]. However, a deep understanding of the role of PVP in the catalytic activity of supported colloidal NPs has not yet been elucidated.

The selective oxidation of bioplatform molecules into value-added chemicals is very important. Aerobic oxidation of 5-hydroxymethylfurfural (HMF), one of the “top” platform bio-chemicals [12,13,14,15,16,17,18,19], can produce many value-added chemicals, including 2,5-diformylfuran (DFF) [20,21,22,23], 5-hydroxymethyl-2-furancarboxylic acid (HMFCA) [24,25,26], and 2,5-furandicarboxylic acid (FDCA) [27,28,29,30,31,32]. HMFCA, an emerging reaction intermediate derived from HMF oxidation, can act as a precursor to synthesize various polyesters [25].

In the past, studies on the oxidation of HMF into HMFCA have been scarce because HMFCA is a reaction intermediate and tends to be further oxidized to FFCA or FDCA, which is catalyzed by noble metal catalysts such as Pt, Au, and Pd NPs [33]. Grunwaldt et al. used Ag/ZrO_2_ catalysts to obtain a high yield of HMFCA up to 98% under a mild condition [26]. Recently, our group also developed an Ag-PVP/ZrO_2_ catalyst to catalyze the HMF oxidation into HMFCA with an excellent yield [8].

Motivated by our recent work demonstrating that PVP can efficiently boost oxidation activity, herein, we used PVP with different MWs to mediate MSI between the Ag NPs and ZrO_2_ support. We also investigated the relationship among MSI, MWs of PVP, and their catalytic properties. To the best of our knowledge, the impact of PVP molecular weight on MSI of supported Ag NP catalysts has not yet been systematically exploited. In addition, a deep understanding of the promotional effect of PVP on the HMF oxidation to HMFCA was revealed.

## 2. Materials and Methods

### 2.1. Synthesis of Catalysts

PVP, AgNO_3_, NaBH_4_, ZrO_2_ were purchased from Aladdin Co. Ltd. (Shanghai, P.R. China) ZrO_2_-supported Ag NPs stabilized by PVP with different MWs were fabricated using an impregnation method. Three types of PVP with different MWs (24, 58, and 130 kDa) were used as the capping agents. For all catalysts, the Ag loading was 2.5 wt % and the Ag/PVP molar ratio was equal to 1. A typical Ag colloidal solution was fabricated according to our previous study [8]. The desired amounts of PVP and AgNO_3_ (an Ag/PVP molar ratio of 1 was used) were added to 100 mL aqueous solution at room temperature, and the solution was stirred for 2 h using a magnetic stirrer. Subsequently, the NaBH_4_ (0.1 mol/L, the NaBH_4_/Ag mole ratio was 4) aqueous solution was slowly added into the solution containing PVP and AgNO_3_ under vigorous stirring. Subsequently, 2 g ZrO_2_ was added and impregnated for 12 h and then water was removed by concentrating in vacuo to obtain various samples with different PVP MWs. The catalysts are named as Ag/ZrO_2_ (MW), where MW refers to the MW of PVP. Two unsupported Ag colloidal catalysts were also fabricated according to the above procedures, except that ZrO_2_ was not added and the impregnation step was not used. An Ag/ZrO_2_ (2.5 wt %) without PVP modification was also prepared via a wet impregnation method.

### 2.2. Characterization of Catalysts

Powder X-ray diffraction (XRD) was recorded on Ultima IV with Cu Kα radiation (λ = 1.5406 Å) at 40 kV and 30 mA at a scanning rate of 5°/min^−1^.

Transmission Electron Microscope analysis was measured using a JEM-2100 transmission electron microscope operating at 200 kV. For the measurement, a small amount of sample was suspended in ethanol and sonicated for a few minutes. Several drops of this suspension were then deposited on a Cu-grid (200 mesh) to dry at room temperature. At least 300 particles were used to statistically calculate the average Ag NP size.

X-ray Photoelectron Spectroscopy (XPS) analyses were performed using a KRATOS AXIS Ultra DLD (Rigaku, Matsubaracho, Japan) using an Al Kα line as the radiation source. The peak area ratio of Ag 3d_5/2_ to 3d_3/2_ of Ag^0^ and Ag_2_O was set up to 3:2 during the deconvolution of the XPS spectra. The binding energy scale was referenced to the C1s line (284.8 eV).

Fourier transform infrared (FT-IR) measurements were measured on a Nicolet 380 FT-IR spectrometer (Nicolet, Waltham, MA, USA) with a spectral resolution of 4 cm^−1^ in the wave number range of 500–4000 cm^−1^. All samples were diluted using the same catalyst:KBr weight ratio (1:50). The mixture was ground in an agate mortar to homogenize it, and then a cylindrical pellet was obtained in a manual mounting press.

### 2.3. Catalytic Reactions and Product Analysis

The oxidation of HMF was performed using a three-necked flask with an oxygen atmosphere under 1 bar pressure at a flowing rate of 60 mL/min as the oxidant. For supported catalysts, the reaction condition was described as follows: 0.20 g HMF, 0.126 g NaOH, 50 mL H_2_O, 0.05 g catalyst, 60 mL/min O_2_, and 30 °C. For unsupported colloidal catalysts, the reaction condition was similar to that used for the supported catalysts, except for the catalyst mass, which contained equal Ag content of the supported catalysts. The products were analyzed by HPLC (Agilent 1200) (Agilent, Palo Alto, CA, USA) equipped with a C_18_ column using 15.0 vol.% CH_3_CN-1.0 vol.‰ CH_3_COOH aqueous solution as a mobile phase. The quantitative analysis was conducted by the external standard method with three parallel measurements. The standard deviations were ± 5%. The total turnover number (TON) was calculated by the converted HMF molecules at the reaction time of 2 h (the unit is mmol/h). The conversion and product yield were calculated according to the following formulas:

(1) $$\mathrm{Conversion}\;\mathrm{of}\;\mathrm{HMF}\;=\;\frac{{\left[\mathrm{HMF}\right]}_{i}-{\left[\mathrm{HMF}\right]}_{f}}{{\left[\mathrm{HMF}\right]}_{i}}\;\times 100$$

(2) $$\mathrm{Yield}\;=\;\frac{{\left[\mathrm{HMFCA}\right]}_{f}}{{\left[\mathrm{HMF}\right]}_{i}}\;\times 100$$

### 2.4. Kinetic Study

The kinetic study on the HMF oxidation was conducted by varying the reaction temperature. The reaction rate constant (*k*) was obtained by the formula, (ln *C*_0_ − ln *C*)/*t*, at a low conversion of HMF considering the oxidation reaction is the first order reaction, where *C*_0_, *C*, *t* refer to the initial HMF concentration, the HMF concentration at the reaction time of t, and the reaction time, respectively. The apparent activation energy (*E_a_*) was calculated by plotting ln *k* versus 1/*T* through the Arrhenius equation.

## 3. Results and Discussion

### 3.1. The Nature of the Catalyst

#### 3.1.1. XRD Results

**Figure 1:**
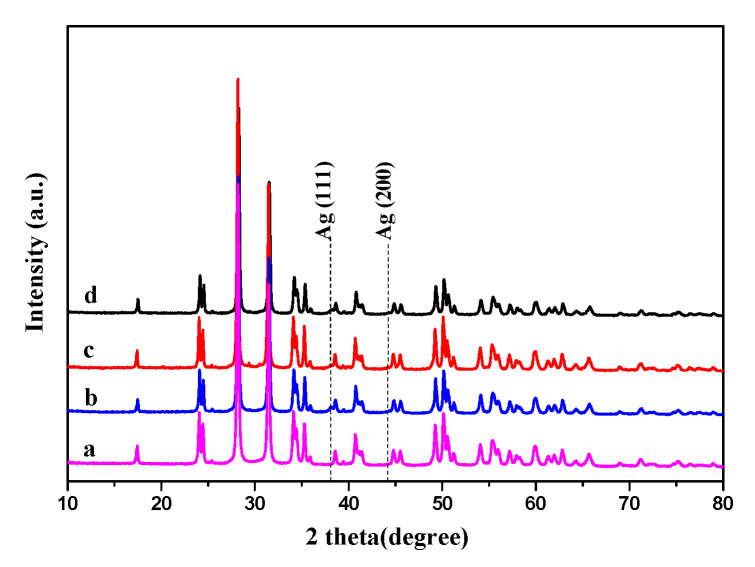
XRD patterns of catalysts: (**a**) ZrO_2_, (**b**) Ag/ZrO_2_ (1,300,000), (**c**) Ag/ZrO_2_ (58,000), and (**d**) Ag/ZrO_2_ (24,000).

Figure 1 illustrates the XRD patterns of the ZrO_2_ support and the Ag NPs catalysts stabilized by PVP with varying MWs. As can be observed, several strong diffraction peaks at 24.1°, 28.2°, 34.2°, 35.4°, and 49.3° appeared for the four catalysts. The diffraction peaks were characteristic of the monoclinic crystal structure of m-ZrO_2_ (PDF. # 37-1484). In addition, two weak signals were also observed at 38.1° and 44.2°, corresponding to the (111) and (200) facets of a Ag NP, respectively [34].

#### 3.1.2. TEM Results

**Figure 2:**
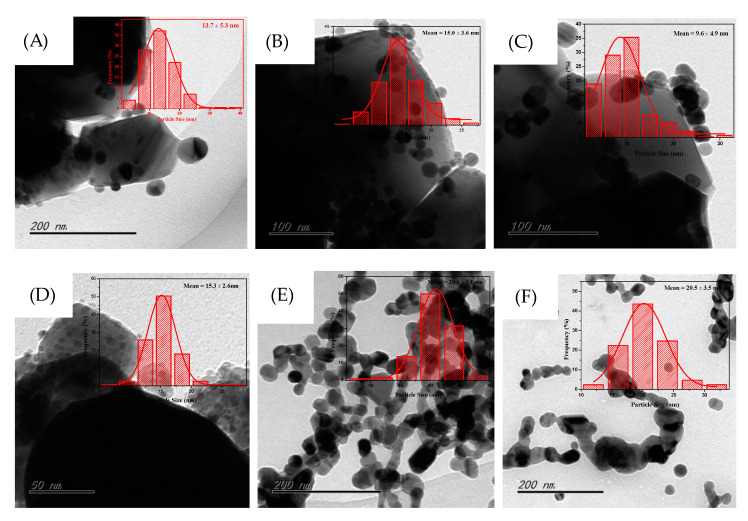
TEM images and particle size distributions: (**A**) Ag/ZrO_2_ (24,000), (**B**) Ag/ZrO_2_ (58,000), (**C**) Ag/ZrO_2_ (1,300,000), (**D**) Ag/ZrO_2_, (**E**) Ag-PVP (58,000), and (**F**) Ag-PVP (1,300,000).

To further measure average Ag particle sizes, a TEM technique was used. Figure 2 presents the TEM images and particle sizes distributions of the supported Ag NP catalyst and the unsupported Ag colloidal catalysts. As can be seen, the mean particle size of Ag/ZrO_2_ (24,000) was approximately 13.7 ± 5.3 nm, while the average particle sizes of Ag/ZrO_2_ (58,000) and Ag/ZrO_2_ (130,000) were approximately 15.0 ± 3.6 nm and 15.3 ± 2.6 nm, respectively. This result means that the average particle size did not change remarkably with the PVP molecular weight. HRTEM images further showed that PVP molecules covered the external surface of Ag NPs for the three PVP-capping catalysts, while no PVP layer was observed for Ag/ZrO_2_ (SI, Figure S1). STEM images of Ag/ZrO_2_ (58,000) demonstrated that surface Ag NPs was composed of Ag^0^. However, for the Ag/ZrO_2_ catalyst without PVP capping agent, the mean particle size was approximately 9.6 ± 4.9 nm.

This phenomenon could be due to the fact that there exists a relatively strong interaction between Ag metal and ZrO_2_. The TEM images further implied that the incorporation of PVP was able to effectively weaken MSI between Ag metal and ZrO_2_. For comparison, the unsupported Ag colloidal catalysts stabilized by PVP were fabricated, and the corresponding TEM results are shown in Figure 2E,F. As can be observed, the Ag NPs aggregated like pearl chains for the two colloidal catalysts, which was completely different from the supported Ag NPs catalyst with high dispersion. The average particle sizes of Ag-PVP (58,000) and Ag-PVP (130,000) were approximately 20.6 ± 3.1 nm and 20.5 ± 3.5 nm, respectively. This observation showed that increases in PVP molecular weight did not change the Ag mean particle sizes. Interestingly, compared to the unsupported Ag colloidal particles, the supported Ag NPs catalysts had a smaller particle size, suggesting that MSI had a remarkable effect on average particle size of Ag NPs.

#### 3.1.3. IR Spectra of the Catalysts

**Figure 3:**
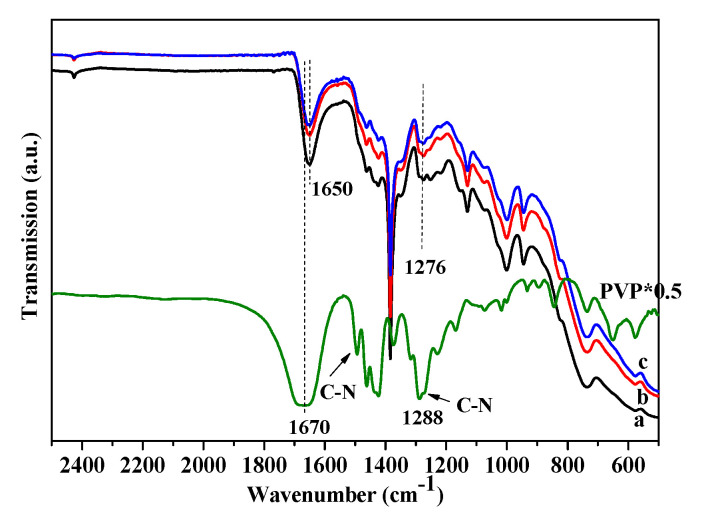
FT-IR spectra of polyvinylpyrrolidone (PVP) and the catalysts: (**a**) Ag/ZrO_2_ (58,000), (**b**) Ag/ZrO_2_ (130,000), and (**c**) Ag/ZrO_2_ (24,000).

Figure 3 illustrates the FT-IR spectra of the supported Ag/ZrO_2_ (MW) capped by PVP with various MWs and a pure PVP. For PVP molecules, the bands at 1286, 1372, 1424, 1461, 1496, and 1670 cm^−1^ were observed. The peak at 1286 cm^−1^ was attributed to the vibration of N–C bonds, whereas the bands between 1300 and 1480 cm^−1^ were related to the typical C–H vibrations of the PVP molecules. The band at 1496 cm^−1^ was attributed to the N–C stretching mode of N–C=O group. A band at 1670 cm^−1^ was related to the stretching mode of C=O [35,36,37].

After the loading of Ag nanoparticles, some changes in the IR spectra were observed, and the Ag-PVP/ZrO_2_ catalysts exhibited several bands at 1276, 1380, 1461, 1488, and 1650 cm^−1^. In comparison with free PVP molecules, the IR spectra of the three supported Ag NPs catalysts exhibited some differences including the band intensity and the shift in the wavenumber, implying that PVP molecules interacted with Ag NPs. The band at 1286 cm^−1^ assigned to the vibration mode of N–C bonds of free PVP molecules shifted to a lower wavenumber at 1276 cm^−1^ for Ag-PVP/ZrO_2_, demonstrating that N atoms interacted with Ag NPs. The band at 1650 cm^−1^ was associated with the CO vibrational mode of the PVP molecule coordinating with the Ag NPs [35,36]. The shift of the carbonyl group from 1670 cm^−1^ to 1650 cm^−1^ can be observed because an interaction occurred between the carbonyl group and surface Ag metal sites.

#### 3.1.4. XPS Results

**Figure 4:**
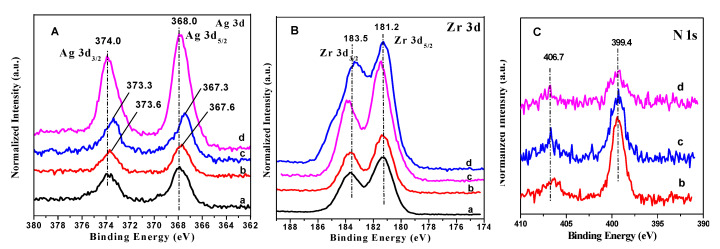
XPS for (**A**) Ag 3d, (**B**) Zr 3d, and (**C**) N 1s of the catalysts: (**a**) Ag/ZrO_2_, (**b**) Ag/ZrO_2_ (24,000), (**c**) Ag/ZrO_2_ (58,000), and (**d**) Ag/ZrO_2_ (1,300,000).

XPS was employed to reveal the effect of PVP MW on the interaction between PVP and ZrO_2_. It can be observed from Figure 4A that the Ag 3d XPS results of the four catalysts were composed of two peaks at approximately 368.0 and 374.0 eV, which were respectively assigned to the 3d electron transitions [38]. Interestingly, after the introduction of PVP, shifts of the binding energies related to the 3d transitions were observed. Moreover, it was found that the binding energy assigned to 3d_3/2_ shifted from 374.0 eV for Ag/ZrO_2_ to 373.6 eV for Ag/ZrO_2_ (24,000), and further shifted to 373.3 eV for Ag/ZrO_2_ (58,000) with increasing PVP molecular weight. However, as the PVP MW further increased to 130,000, no obvious shift was observed for Ag/ZrO_2_ (1,300,000). These results clearly demonstrated that PVP MW had a profound effect on the electronic structure of the catalysts. The downshift in binding energies attributed to the Ag 3d electron was indicative of an electron transfer from PVP to Ag NPs. It should be noted that a portion of the Ag^0^ metal of Ag NPs was oxidized to Ag_2_O after exposure to air according to the deconvoluted spectral results (see Supplementary Materials, Figure S3).

Figure 4B illustrates the Zr 3d XPS spectra of the samples. Two signals at 181.2 and 183.5 eV appeared, which were assigned to the 3d electron transitions [39], respectively. Upshifts of the binding energies for Zr 3d electrons were also observed for Ag/ZrO_2_ (24,000) and Ag/ZrO_2_ (58,000), which indicated CT from ZrO_2_ to Ag^0^ [39]. However, for Ag/ZrO_2_ (1,300,000), no obvious shift for Zr 3d_5/2_ was observed.

To gain insights into MSI between Ag NPs and PVP, the N 1s spectra are presented in Figure 4C. From the figure, two bands appear at 399.4 and 406.7 eV. The peak at 399.4 eV is associated with PVP, implying that part of PVP molecules could weakly coordinate with the Ag NP surfaces [40]. The other N 1s peaks at 406.7 eV is unidentified, which could be a species derived from the cleavage of pyrrolidone rings and subsequently hydrolysis, anchored on different Ag metal facets. Based on the N 1s XPS results, we propose that PVP chemisorbs onto the Ag NPs surfaces mainly via the nitrogen atom or the oxygen atom of the pyrrolidone ring.

### 3.2. Oxidation of HMF to HMFCA

In order to evaluate the catalytic activity of these samples, aerobic oxidation of HMF was undertaken. It should be noted that only HMFCA and minor humins were detected for the oxidizing products, and no other oxidation products such as DFF, FFCA, or FDCA were detected. For comparative purposes, two blank reactions without catalyst or by using ZrO_2_ were also conducted. The reaction results showed that the oxidation activity of HMF could be negligible in the two blank reactions.

**Figure 5:**
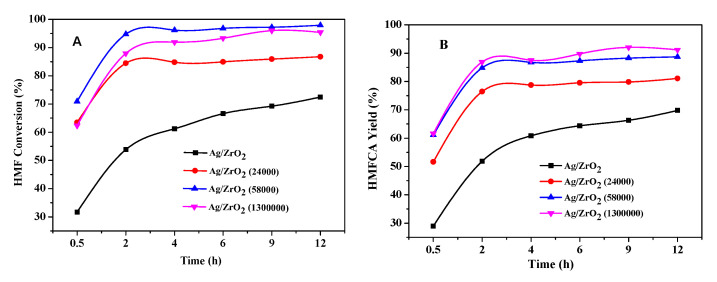
5-hydroxymethylfurfural (HMF) conversion (**A**) and 5-hydroxymethyl-2-furancarboxylic acid (HMFCA) yield (**B**) evolved with the reaction time on supported Ag NP catalysts. Reaction conditions: 0.20 g HMF, 0.126 g NaOH, 50 mL H_2_O, 0.05 g catalyst, 60 mL/min O_2_, 30 °C.

Figure 5 shows the HMF conversion and product yield as a function of reaction times. It can be observed that the incorporation of PVP substantially increased the HMF conversion and HMFCA yield on the three PVP-stabilizing Ag/ZrO_2_ catalysts, compared with Ag/ZrO_2_. In order to further compare their catalytic activities, TON of each catalyst was calculated based on the HMF conversion at the reaction time of 2 h. The corresponding TON value decreased in the order Ag/ZrO_2_ (58,000) (0.75 mmol/h) > Ag/ZrO_2_ (1,300,000) (0.70 mmol/h) > Ag/ZrO_2_ (24,000) (0.67 mmol/h) > Ag/ZrO_2_ (0.43 mmol/h). It should be noted that Ag/ZrO_2_ catalyst presents a minimum size (~9.6 nm) among the supported catalysts, but it exhibited inferior reaction activity, which is because stronger MSI has a negative impact on its oxidizing activity. In addition, Ag/ZrO_2_ (58,000) exhibited the highest HMF conversion, but Ag/ZrO_2_ (1,300,000) afforded higher selectivity toward HMFCA even though the two catalysts have a similar average particle size (15.0 nm vs. 15.3 nm). We speculate that an increase in the MW of PVP enlarges the distance between Ag and ZrO_2_, thereby weakening their interaction. It seems that the weak MSI favors the HMF oxidation. However, Ag/ZrO_2_ (1,300,000) did not exhibit the highest activity, which could be due to the fact that Ag/ZrO_2_ (1,300,000) has the most substantial steric hindrance effects because the MW of PVP is the largest among the three Ag-PVP/ZrO_2_ catalysts.

**Figure 6:**
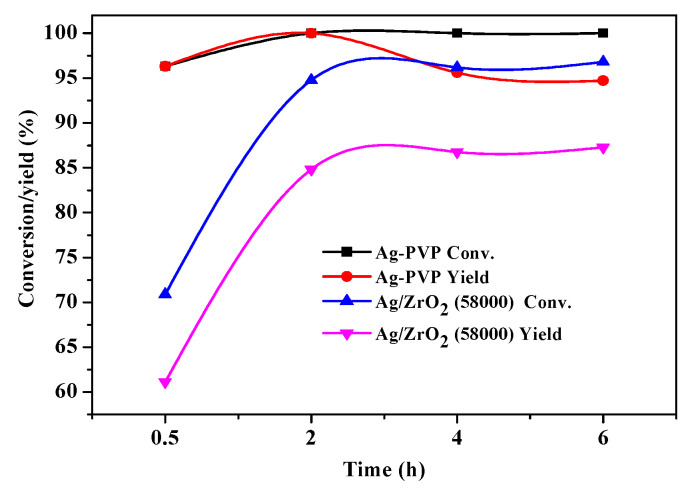
HMF conversion and HMFCA yield as functions of time on Ag/ZrO_2_ (58,000) and unsupported colloidal Ag-PVP (58,000). Reaction conditions: 0.20 g HMF, 0.126 g NaOH, 50 mL H_2_O, 60 mL/min O_2_, and 30 °C.

We also explored the impact of the ZrO_2_ support on the HMF oxidation, and the corresponding results are shown in Figure 6. Compared with Ag/ZrO_2_ (58,000), the unsupported Ag-PVP colloidal catalyst produced a significantly higher catalytic activity, and the HMF conversion reached 95% even with a very short reaction time, but the HMFCA yield was reduced with the reaction time as a consequence of side reactions. The corresponding TON values, which were calculated on the basis of the HMF conversion at the reaction time of 0.5 h, were 2.25 mmol/h and 3.05 mmol/h for Ag/ZrO_2_ (58,000) and unsupported colloidal Ag-PVP (58,000), respectively. These results further show that the metal–support interaction had a crucial effect on the HMF oxidation.

### 3.3. Reaction Kinetics

The kinetic study on the oxidation of HMF was performed by changing the reaction temperature. Three catalysts, i.e., Ag/ZrO_2_, Ag/ZrO_2_ (58,000), and unsupported Ag-PVP (58,000), were selected to further elucidate the influence of PVP on the HMF oxidation. To assure the low conversion of HMF, the reaction time was less than 15 min. The apparent activation energy (*Ea*) values of Ag/ZrO_2_, Ag/ZrO_2_ (58,000), and unsupported Ag-PVP (58,000) were estimated to be 29.8, 12.8, and 9.0 kJ/mol (SI, Figure S6), respectively. It has been demonstrated that MSI could remarkably modify adsorption energies and thus influence the reaction kinetics [41]. One possible explanation for this result is that the introduction of PVP could efficiently weaken the interaction of Ag NPs with ZrO_2_ by increasing their distance and changing the electronic effect of Ag NPs, thereby affecting the adsorption and activation of HMF on these catalysts.

### 3.4. The Relationship Between Metal–Support Interaction and PVP Molecular Weight

In our previous work, it was found that the presence of PVP can efficiently boost the catalytic performance in the HMF oxidation owing to the modulation of MSI [8]. In this work, the Ag/ZrO_2_ catalyst (~9.6 nm) did not show superior oxidation activity compared to the other three PVP-capping Ag catalysts (~15.0 nm). In addition, we found that the PVP MW had a significant influence on the oxidation activity owing to electronic effects, as evidenced by the XPS results (Figure 4).

Based on the characterized data and the HMF oxidation results of the catalysts, we propose that PVP molecules absorb on surface Ag metal sites to form a strong interfacial interaction, which could efficiently alter the interaction between Ag NPs and ZrO_2_. This proposal is confirmed by the XPS results, indicating that PVP MW can dramatically result in up-/down-shifting of the binding energies of Ag 3d and Zr 3d electrons (Figure 4). Considering the aforementioned results, superior catalytic activities of Ag/ZrO_2_ capped by PVP could be attributed to a high negative charge on the Ag NP surfaces. The binding energy of Ag 3d electrons relies on charge transfers (CT) between Ag and ZrO_2_, together with that between Ag and PVP molecules, which depends on the coordination situations of the N and/or O atoms. We hypothesize that with increasing PVP molecular weight, the distance between Ag and ZrO_2_ might increase, thereby reducing their CT. This was corroborated by XPS results indicating that a shift of the Ag 3d binding energy occurred with changing PVP MW.

## 4. Conclusions

In summary, we have developed a novel strategy to precisely tune the metal–support interaction of the ZrO_2_-supported Ag NPs catalysts via changing MW of the capping agent-PVP. The polymer can remarkably enhance the activity of HMF oxidation, because PVP is able to efficiently tune the electronic configuration of Ag NPs by charge transfers among Ag NPs, PVP, and ZrO_2_ support, but further increasing MW (i.e., 130,000) could lead to a decrease in the oxidation activity due to steric hindrance effects. A proper metal–support interaction could increase the electronegativity of surface Ag sites by modulating the charge transfers among Ag NPs, ZrO_2_ support, and PVP. However, a deeper understanding of the interactions among the support, the stabilizing agent, and Ag active sites is needed, and some more advanced techniques, together with DFT studies, are required to promote research in the field.

## Supplementary Materials

The following are available online at https://www.mdpi.com/2079-4991/10/9/1624/s1, Figure S1: X-ray photoelectron spectra for Ag 3d and Zr 3d of the 2.5%Ag/ZrO_2_ catalysts, Figure S2 XPS for N 1s of (A) 2.5%Ag-PVP/ZrO_2_ (24,000), (B) 2.5%Ag-PVP/ZrO_2_ (58,000), and (C) 2.5%Ag-PVP/ZrO_2_ (1,300,000), Figure S3 X-ray photoelectron spectra for O 1s of (A) 2.5%Ag-PVP/ZrO_2_ (24,000), (B) 2.5%Ag-PVP/ZrO_2_ (58,000), (C) 2.5%Ag-PVP/ZrO_2_ (1,300,000); (D) 2.5%Ag/ZrO_2_, Figure S4 Plot of lnk versus 1/T. Reaction conditions: 0.2 g HMF, 0.126 g NaOH, 50 mL H_2_O, 0.05 g catalyst (for 2.5%Ag/ZrO_2_ and 2.5%Ag-PVP/ZrO_2_ (58,000), 0.01 g catalyst (for 2.5%Ag-PVP (58,000)), 60 mL/min O_2_, 30 °C.

## References

[1] Jackson C., Smith G.T., Inwood D.W., Leach A., Whalley P.S., Callisti M., Polcar T., Russell A.E., Levecque P.B.J., Kramer D. (2017). Electronic metal–support interaction enhanced oxygen reduction activity and stability of boron carbide supported platinum. Nat. Commun..

[2] Rossi L.M., Fiorio J.L., Garcia M.A.S., Ferraz C.P. (2018). The role and fate of capping ligands in colloidally prepared metal nanoparticle catalysts. Dalton Trans..

[3] Matsubu J.C., Zhang S., Derita L., Marinković N., Chen J.G., Graham G.W., Pan X., Christopher P. (2016). Adsorbate-mediated strong metal–support interactions in oxide-supported Rh catalysts. Nat. Chem..

[4] Jiang Z.-S., Zhao Y.-H., Huang C.-F., Song Y.-H., Li D.-P., Liu Z.-T., Liu Z.-W. (2018). Metal–support interactions regulated via carbon coating—A case study of Co/SiO_2_ for Fischer-Tropsch synthesis. Fuel.

[5] Oh S., Kim Y.K., Jung C.H., Doh W.H., Park J.Y. (2018). Effect of the metal–support interaction on the activity and selectivity of methanol oxidation over Au supported on mesoporous oxides. Chem. Commun..

[6] Murata K., Mahara Y., Ohyama J., Yamamoto Y., Arai S., Satsuma A. (2017). The Metal–support Interaction Concerning the Particle Size Effect of Pd/Al_2_O_3_ on Methane Combustion. Angew. Chem. Int. Ed..

[7] Wang L., Zhang J., Zhu Y., Xu S., Wang C., Bian C., Meng X., Xiao F.-S. (2017). Strong Metal–Support Interactions Achieved by Hydroxide-to-Oxide Support Transformation for Preparation of Sinter-Resistant Gold Nanoparticle Catalysts. ACS Catal..

[8] An J., Sun G., Xia H. (2019). Aerobic Oxidation of 5-Hydroxymethylfurfural to High-Yield 5-Hydroxymethyl-2-furancarboxylic Acid by Poly(vinylpyrrolidone)-Capped Ag Nanoparticle Catalysts. ACS Sustain. Chem. Eng..

[9] Sun G., An J., Hu H., Li C., Zuo S., Xia H. (2019). Green catalytic synthesis of 5-methylfurfural by selective hydrogenolysis of 5-hydroxymethylfurfural over size-controlled Pd nanoparticle catalysts. Catal. Sci. Technol..

[10] Tsuji M., Nishizawa Y., Matsumoto K., Kubokawa M., Miyamae N., Tsuji T. (2006). Effects of chain length of polyvinylpyrrolidone for the synthesis of silver nanostructures by a microwave-polyol method. Mater. Lett..

[11] Haesuwannakij S., Kimura T., Furutani Y., Okumura K., Kokubo K., Sakata T., Yasuda H., Yakiyama Y., Sakurai H. (2017). The Impact of the Polymer Chain Length on the Catalytic Activity of Poly(N-vinyl-2-pyrrolidone)-supported Gold Nanoclusters. Sci. Rep..

[12] Gorbanev Y.Y., Kegnæs S., Riisager A. (2011). Effect of Support in Heterogeneous Ruthenium Catalysts Used for the Selective Aerobic Oxidation of HMF in Water. Top. Catal..

[13] Li C., Zhao Z.K., Cai H., Wang A., Zhang T. (2011). Microwave-promoted conversion of concentrated fructose into 5-hydroxymethylfurfural in ionic liquids in the absence of catalysts. Biomass Bioenergy.

[14] Tong X., Ma Y., Li Y. (2010). Biomass into chemicals: Conversion of sugars to furan derivatives by catalytic processes. Appl. Catal. A Gen..

[15] Xia H., Xu S., Yan X., Zuo S. (2016). High yield synthesis of 5-hydroxymethylfurfural from cellulose using FePO_4_ as the catalyst. Fuel Process. Technol..

[16] Xia H., Xu S., Yang L. (2017). Efficient conversion of wheat straw into furan compounds, bio-oils, and phosphate fertilizers by a combination of hydrolysis and catalytic pyrolysis. RSC Adv..

[17] Yang L., Yan X., Wang Q., Wang Q., Xia H. (2015). One-pot catalytic conversion of cellulose into polyols with Pt/CNTs catalysts. Carbohydr. Res..

[18] Xu S., Pan D., Hu F., Wu Y., Wang H., Chen Y., Yuan H., Gao L., Xiao G. (2019). Highly efficient Cr/beta zeolite catalyst for conversion of carbohydrates into 5-hydroxymethylfurfural: Characterization and performance. Fuel Process. Technol..

[19] Xu S., Yin C., Pan D., Hu F., Wu Y., Miao Y., Gao L., Xiao G. (2019). Efficient conversion of glucose into 5-hydroxymethylfurfural using a bifunctional Fe^3+^ modified Amberlyst-15 catalyst. Sustain. Energy Fuels.

[20] Biswas S., Dutta B., Mannodi-Kanakkithodi A., Clarke R., Song W., Ramprasad R., Suib S.L. (2017). Heterogeneous mesoporous manganese/cobalt oxide catalysts for selective oxidation of 5-hydroxymethylfurfural to 2,5-diformylfuran. Chem. Commun..

[21] Mishra D.K., Cho J.K., Kim Y.J. (2018). Facile production of 2,5-diformylfuran from base-free oxidation of 5-hydroxymethyl furfural over manganese–cobalt spinels supported ruthenium nanoparticles. J. Ind. Eng. Chem..

[22] Ning L., Liao S., Sun Y., Yu L., Tong X. (2016). The Efficient Oxidation of Biomass-Derived 5-Hydroxymethyl Furfural to Produce 2,5-Diformylfuran Over Supported Cobalt Catalysts. Waste Biomass-Valorization.

[23] Hong M., Min J., Wu S., Cui H., Zhao Y., Li J., Wang S. (2019). Metal Nitrate Catalysis for Selective Oxidation of 5-Hydroxymethylfurfural into 2,5-Diformylfuran under Oxygen Atmosphere. ACS Omega.

[24] Zhang Z., Liu B., Lv K., Sun J., Deng K. (2014). Aerobic oxidation of biomass derived 5-hydroxymethylfurfural into 5-hydroxymethyl-2-furancarboxylic acid catalyzed by a montmorillonite K-10 clay immobilized molybdenum acetylacetonate complex. Green Chem..

[25] Wang F., Zhang Z. (2017). Cs-substituted tungstophosphate-supported ruthenium nanoparticles: An effective catalyst for the aerobic oxidation of 5-hydroxymethylfurfural into 5-hydroxymethyl-2-furancarboxylic acid. J. Taiwan Inst. Chem. Eng..

[26] Schade O.R., Kalz K.F., Neukum D., Kleist W., Grunwaldt J.-D. (2018). Supported gold- and silver-based catalysts for the selective aerobic oxidation of 5-(hydroxymethyl)furfural to 2,5-furandicarboxylic acid and 5-hydroxymethyl-2-furancarboxylic acid. Green Chem..

[27] Ardemani L., Cibin G., Dent A.J., Isaacs M., Kyriakou G., Lee A.F., Parlett C.M.A., Parry S.A., Wilson K. (2015). Solid base catalysed 5-HMF oxidation to 2,5-FDCA over Au/hydrotalcites: Fact or fiction?. Chem. Sci..

[28] Li S., Su K., Li Z., Cheng B. (2016). Selective oxidation of 5-hydroxymethylfurfural with H_2_O_2_ catalyzed by a molybdenum complex. Green Chem..

[29] Van Nguyen C., Liao Y.-T., Kang T.-C., Chen J.E., Yoshikawa T., Nakasaka Y., Masuda T., Wu K.C.-W. (2016). A metal-free, high nitrogen-doped nanoporous graphitic carbon catalyst for an effective aerobic HMF-to-FDCA conversion. Green Chem..

[30] Zhang Z., Deng K. (2015). Recent Advances in the Catalytic Synthesis of 2,5-Furandicarboxylic Acid and Its Derivatives. ACS Catal..

[31] Xia H., An J., Hong M., Xu S., Zhang L., Zuo S. (2018). Aerobic oxidation of 5-hydroxymethylfurfural to 2,5-difurancarboxylic acid over Pd-Au nanoparticles supported on Mg-Al hydrotalcite. Catal. Today.

[32] Liu K.-J., Zeng T.-Y., Zeng J.-L., Gong S.-F., He J.-Y., Lin Y.-W., Tan J.-X., Cao Z., He W.-M. (2019). Solvent-dependent selective oxidation of 5-hydroxymethylfurfural to 2,5-furandicarboxylic acid under neat conditions. Chin. Chem. Lett..

[33] Casanova O., Iborra S., Corma A. (2009). Biomass into Chemicals: Aerobic Oxidation of 5-Hydroxymethyl-2-furfural into 2,5-Furandicarboxylic Acid with Gold Nanoparticle Catalysts. ChemSusChem.

[34] Nossova L., Caravaggio G., Couillard M., Ntais S. (2018). Effect of preparation method on the performance of silver-zirconia catalysts for soot oxidation in diesel engine exhaust. Appl. Catal. B Environ..

[35] García-Aguilar J., Navlani-García M., Berenguer-Murcia A., Mori K., Kuwahara Y., Yamashita H., Cazorla-Amoros D. (2016). Evolution of the PVP–Pd Surface Interaction in Nanoparticles through the Case Study of Formic Acid Decomposition. Langmuir.

[36] Evangelisti C., Panziera N., D’Alessio A., Bertinetti L., Botavina M., Vitulli G. (2010). New monodispersed palladium nanoparticles stabilized by poly-(N-vinyl-2-pyrrolidone): Preparation, structural study and catalytic properties. J. Catal..

[37] Xian J., Hua Q., Jiang Z., Ma Y., Huang W. (2012). Size-Dependent Interaction of the Poly(N-vinyl-2-pyrrolidone) Capping Ligand with Pd Nanocrystals. Langmuir.

[38] Boukhvalov D.W., Zhidkov I.S., Kurmaev E.Z., Fazio E., Cholakh S.O., D’Urso L. (2018). Atomic and electronic structures of stable linear carbon chains on Ag-nanoparticles. Carbon.

[39] Zhang X., Wei C., Song Y., Song X., Sun Z. (2014). Nanoporous Ag–ZrO_2_ composites prepared by chemical dealloying for borohydride electro-oxidation. Int. J. Hydrog. Energy.

[40] Collins G., Schmidt M., McGlacken G.P., O’Dwyer C., Holmes J.D. (2014). Stability, Oxidation, and Shape Evolution of PVP-Capped Pd Nanocrystals. J. Phys. Chem. C.

[41] Jenness G.R., Schmidt J.R. (2013). Unraveling the Role of Metal–Support Interactions in Heterogeneous Catalysis: Oxygenate Selectivity in Fischer–Tropsch Synthesis. ACS Catal..

