# Supplementary material for: Aerobic Oxidation of 5-Hydroxymethylfurfural over Ag Nanoparticle Catalysts Stabilized by Polyvinylpyrrolidone with Different Molecular Weights
Source: Nanomaterials (Basel). 2020 Aug 19;10(9):1624. doi: 10.3390/nano10091624 (PMC7558134; doi:10.3390/nano10091624)
Supplement: Supplementary file 1 [file nanomaterials-10-01624-s001.pdf]

Supplementary material for

Aerobic oxidation of 5-hydroxymethylfurfural over Ag nanoparticle catalysts stabilized by polyvinylpyrrolidone with different chain length

Haian Xia*, Jiahuan An, Weizi Zhang

Jiangsu provincial key lab for the chemistry and utilization of agro-forest biomass, College of Chemical Engineering, Nanjing Forestry University, Nanjing 210037, China

Jiangsu Co-Innovation Center of Efficient Processing and Utilization of Forest Resources, Nanjing Forestry University, Nanjing 210037, China

*Corresponding author: Haian Xia

Tel: +86-25-85427635; Fax: +86-25-85428873

E-mail: haxia@dicp.ac.cn

(1) The HMF oxidation setup

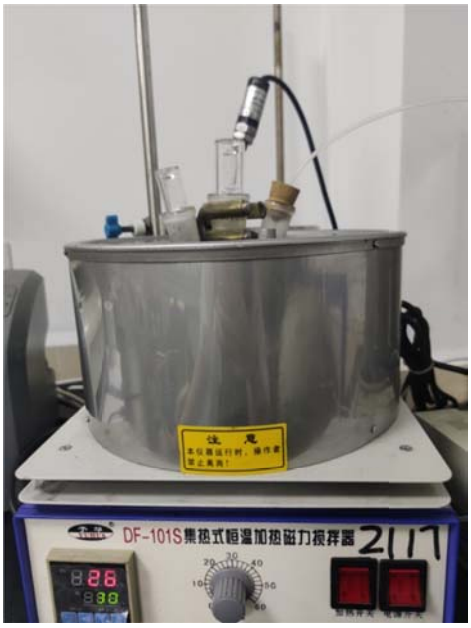

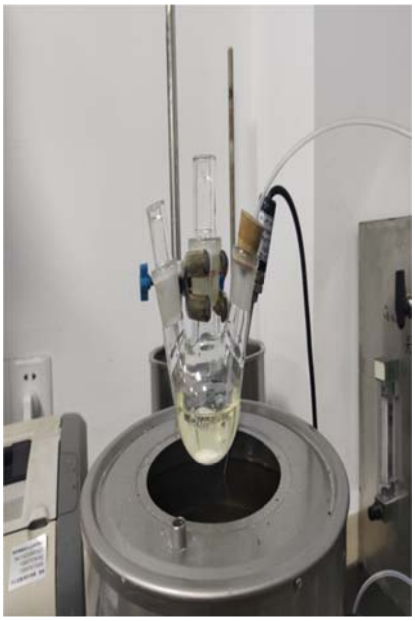

(2) TEM images of the catalysts

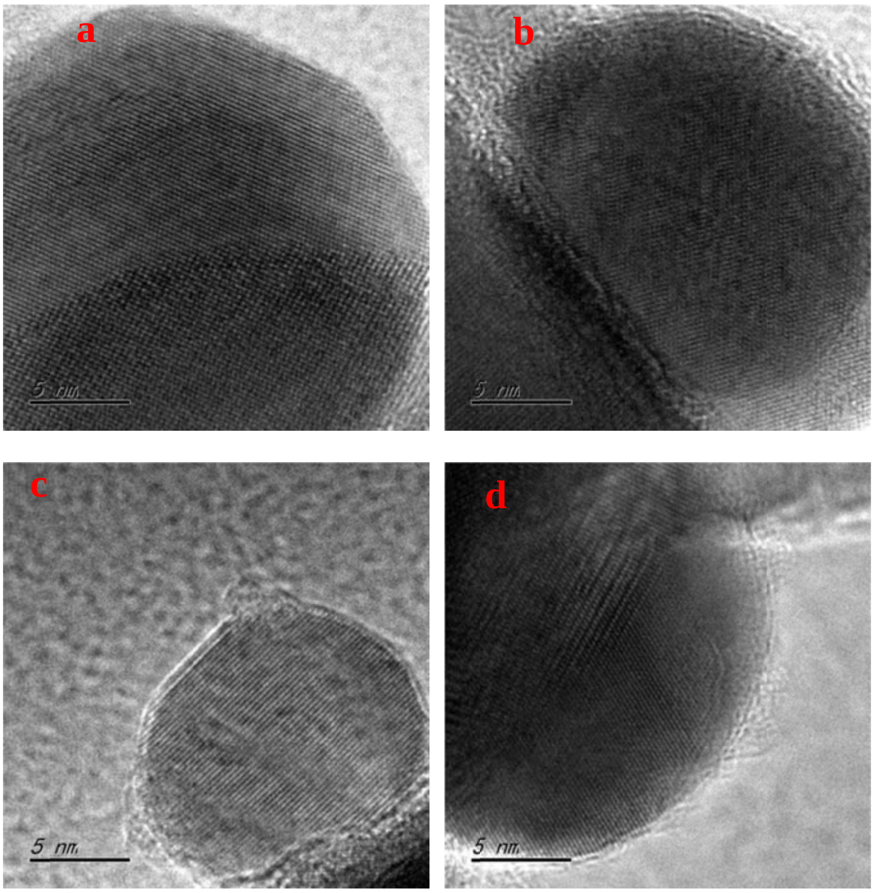

Figure S1 TEM image of (a) Ag/ZrO_2, (b) Ag/ZrO_2(24000), (c) Ag/ZrO_2(58000), and (d)

Ag/ZrO_2(1300000).

（3）*STEM analysis*

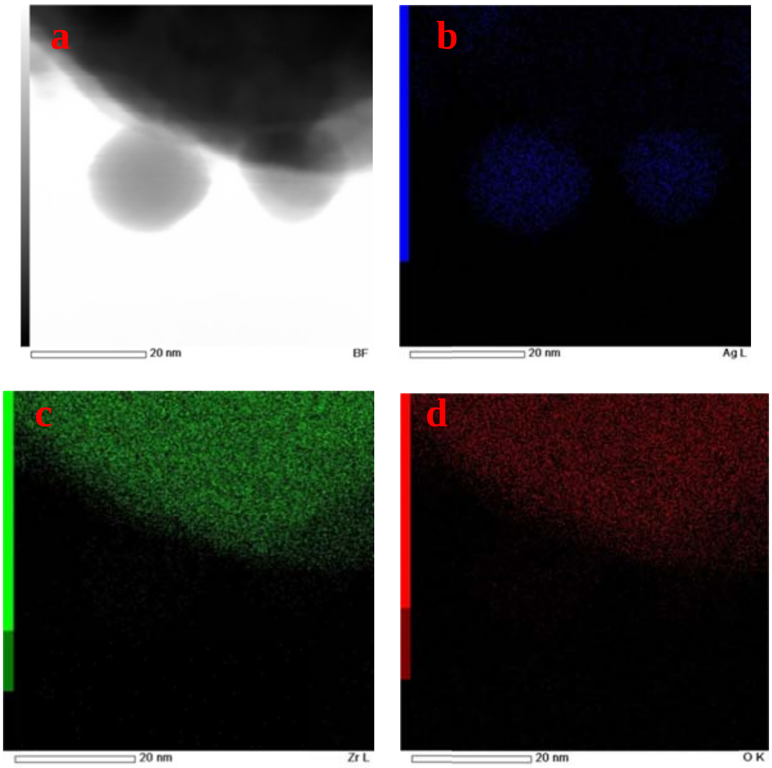

Figure S2 STEM image (a) of and EDX mapping of Ag /ZrO_2(58000) (b: Ag, c: Zr, d: O)

(4) Ag 3d XPS results

Figure S3 illustrates XPS results for Ag 3d of the 2.5%Ag/ZrO_2 catalysts. From Fig. S3, the Ag 3d XPS spectrum is composed of two bands corresponding to the $3d_{3/2}$ and $3d_{5/2}$ transitions, which can each be deconvoluted into two different peaks (a total of 4 peaks): at 367.2 eV and 373.2 eV, assigned to the sliver oxides Ag_2O, at 374.0 eV and 368.0 eV, corresponding to the Ag^0 metal [1, 2]. This means that part of Ag(0) species was oxidized to Ag_2O after exposure to air. The Zr 3d spectrum is consisted of

two doublet with $3d_{5/2}$ and $3d_{3/2}$ located at 181.4 eV and 183.7 eV, respectively, corresponding to Zr^{4+}[3]. For other samples, similar deconvoluted results wereobtained but the percentages of Ag^0 and Ag_2O are different (the result not shown).

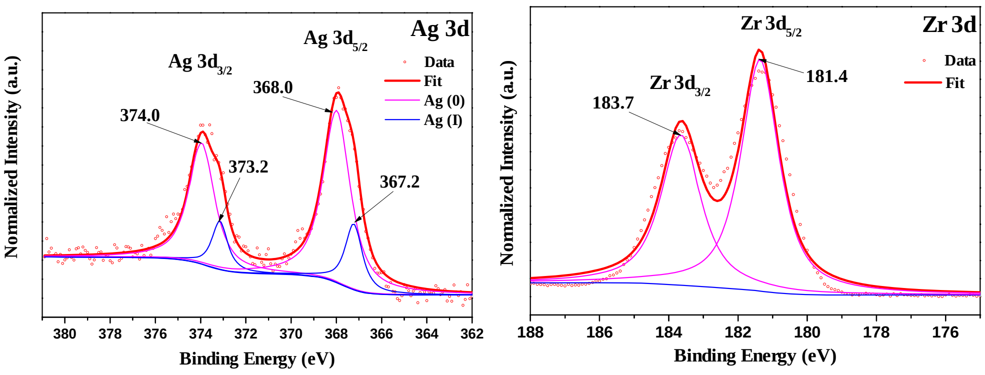

Figure S3 X-ray photoelectron spectra for Ag 3d and Zr 3d of the Ag/ZrO_2 catalysts

To provide a better insight into Ag metal-PVP interaction, the N 1s spectra are shown in Fig. S4. It can be seen from Fig. S3 that a N 1s binding energy is located at 399.4 eV, which is ascribed to the pyrrolidone N group [4]. The N 1s binding energy at 399.4 eV is similar to that of the free PVP, suggesting that some N atoms do not bind to or weakly anchor on the Ag NPs surface. Besides 399.4 eV, the other N 1s binding energy are observed at 406.5 eV (Fig. S4). Shifts to a higher binding energy are attributed to decreased electron density of the N group. Huang et al. concluded that the chemisorption of PVP to spherical Pd NPs can also crack the N-C bond in the N-C=O group, with subsequent hydrolysis produce CH_2-CH_2-NH_2^+-$(CH_2)_3$-COO^-[5]. Based on the N 1s spectral results that a high binding energy at 406.5 eV appears, we hypothesis that some PVP-rings were subject to ring breakage. The binding energy at

406.5 eV is unidentified and might be ascribed to more positively charged amine species anchored on different Ag metal facet. According to N 1s XPS results, the PVP bought to the Ag metal NPs surface mainly through the O atom and N atom of pyrrolidone cleavage ring. A similar phenomenon was also observed for the other Ag NPs catalysts (Fig. S4).

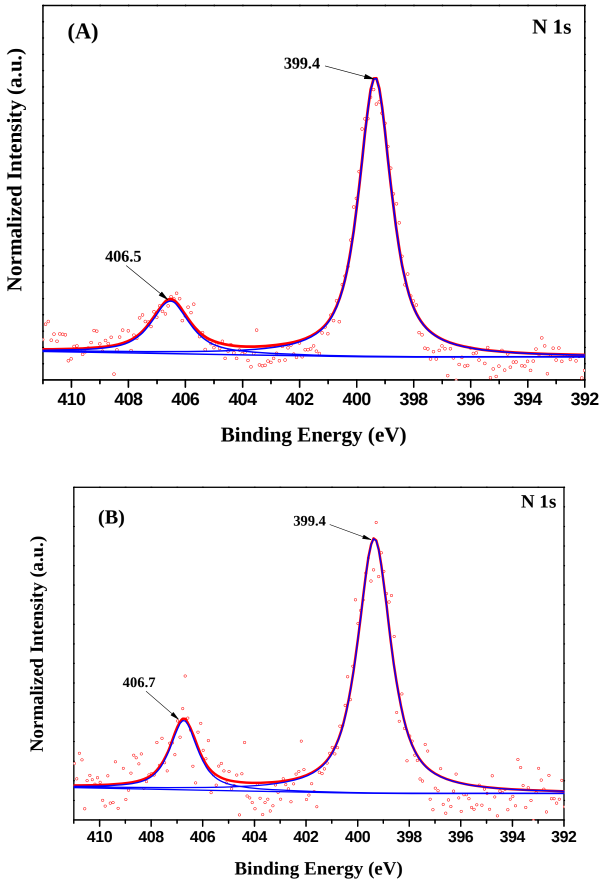

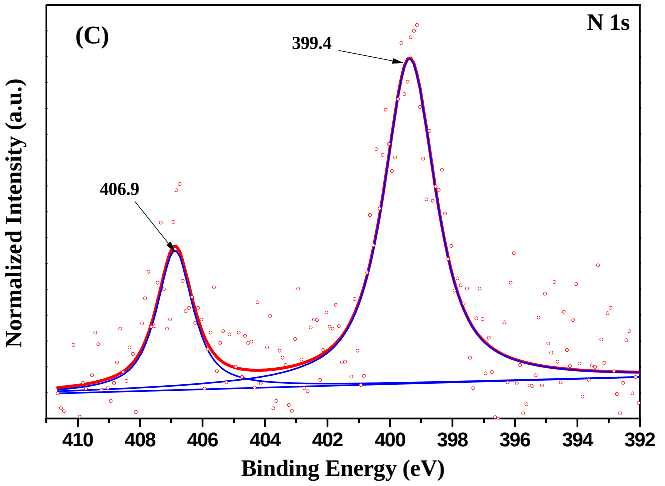

Figure S4 XPS for N 1s of (A) Ag/ZrO_2(24000), (B) Ag /ZrO_2(58000), and (C) Ag /ZrO_2(1300000).

Figure S5 shows the X-ray photoelectron spectra for O 1s of the four catalysts. Three peaks can be observed for the four samples. The binding energies at ~532.4 and ~530.0 eV are related to ZrO_2 support [6], while the peak at ~529.1 eV is assigned to surface oxygen species on Ag NPs.

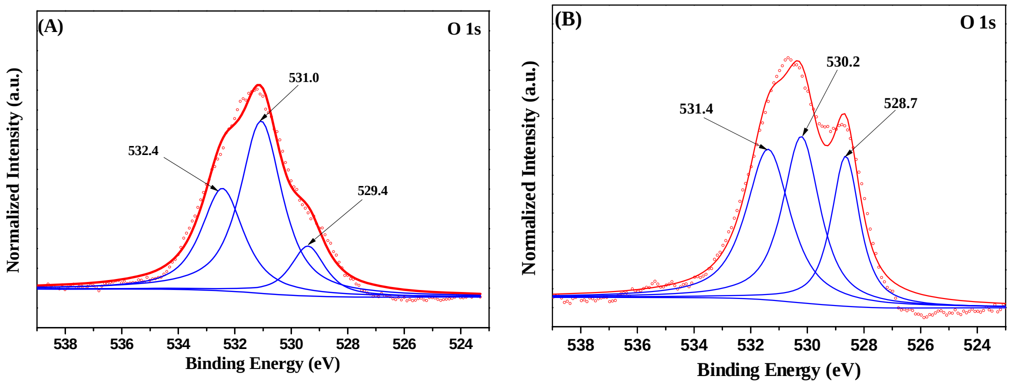

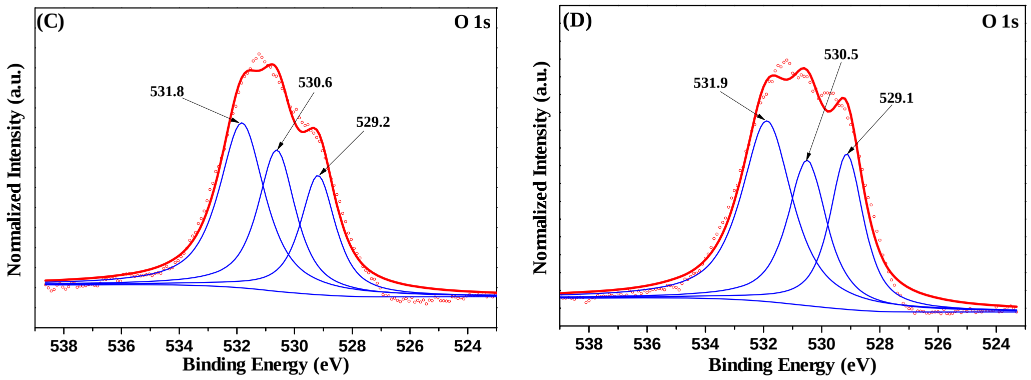

Figure S5 X-ray photoelectron spectra for O 1s of (A) 2.5%Ag-PVP/ZrO_2(24000), (B)2.5%Ag-PVP/ZrO_2(58000), (C) 2.5%Ag-PVP/ZrO_2(1300000); (D) 2.5%Ag/ZrO_2.

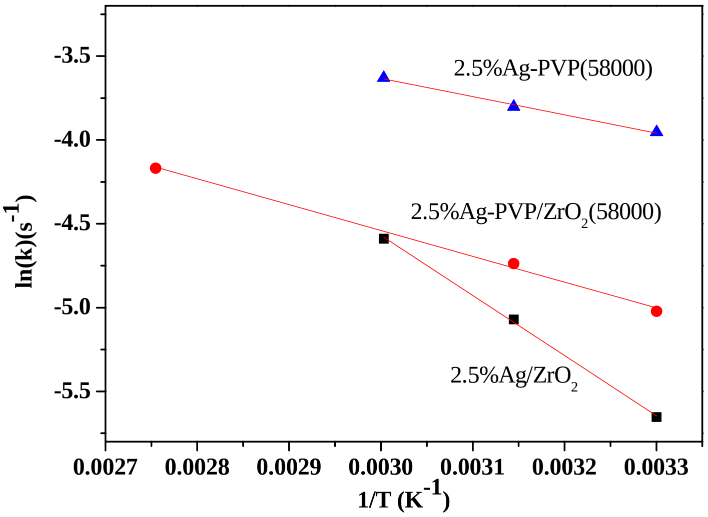

Figure S6 Plot of lnk versus 1/T. Reaction conditions: 0.2 g HMF, 0.126 g NaOH, 50 mL H_2O, 0.05 g catalyst (for 2.5%Ag/ZrO_2 and 2.5%Ag-PVP/ZrO_2(58000), 0.01 g catalyst (for 2.5%Ag-PVP(58000)), 60 mL/min O_2, 30 °C.

References

[1] Bansala T.; Mukhopadhyay S.; Joshi M.; Doong R.-a.; Chaudhary M. Synthesis and shielding properties of PVP-stabilized-AgNPs-based graphene nanohybrid in the Ku band, Synthetic Metals **2016**,

221, 86-94.
[2] Boukhvalov D.W.; Zhidkov I.S.; Kurmaev E.Z.; Fazio E.; Cholakh S.O.; D'Urso L. Atomic and electronic structures of stable linear carbon chains on Ag-nanoparticles, Carbon **2018**, *128*, 296-301.
[3] Zhang X.; Wei C.; Song Y.; Song X.; Sun Z. Nanoporous Ag-ZrO2 composites prepared by chemical dealloying for borohydride electro-oxidation, International Journal of Hydrogen Energy **2014**, *39*, 15646-55.
[4] Collins G.; Schmidt M.; McGlacken G.P.; O'Dwyer C.; Holmes J.D. Stability, Oxidation, and Shape Evolution of PVP-Capped Pd Nanocrystals, The Journal of Physical Chemistry C **2014**, *118*, 6522-30.
[5] Xian J.; Hua Q.; Jiang Z.; Ma Y.; Huang W. Size-dependent interaction of the poly(N-vinyl-2-pyrrolidone) capping ligand with Pd nanocrystals, Langmuir **2012**, *28*, 6736-41.
[6] Patel M.H.; Chaudhuri T.K.; Shripathi T.; Deshpande U.; Patel V.K. Influence of Pb+2-Thiourea complex concentration on the structural, optical, thermal and electrical properties of PbS/PVP-PVA nanocomposite films, Journal of Polymer Research **2017**, *25*.
